# Accurate modeling of high-repetition rate ultrashort pulse amplification in optical fibers

**DOI:** 10.1038/srep34742

**Published:** 2016-10-07

**Authors:** Robert Lindberg, Peter Zeil, Mikael Malmström, Fredrik Laurell, Valdas Pasiskevicius

**Affiliations:** 1 Royal Institute of Technology, Applied Physics, Stockholm, 106 91, Sweden

## Abstract

A numerical model for amplification of ultrashort pulses with high repetition rates in fiber amplifiers is presented. The pulse propagation is modeled by jointly solving the steady-state rate equations and the generalized nonlinear Schrödinger equation, which allows accurate treatment of nonlinear and dispersive effects whilst considering arbitrary spatial and spectral gain dependencies. Comparison of data acquired by using the developed model and experimental results prove to be in good agreement.

High-power ultrafast laser sources are extensively used in applications ranging from pump sources in nonlinear frequency conversion^1^,^2^,^3^,^4^ to metrology^5^,^6^,^7^ and material processing^8^,^9^,^10^,^11^. Master oscillator power amplifier (MOPA) sources based on fiber amplifiers (FA) are especially interesting due to large gain, small foot-print, robustness and efficient heat removal, which alleviates substantially the mode quality degradation brought about by thermo-optic effects at high average powers^12^. The complexity of fiber based MOPA systems vary greatly depending on applications. Some schemes employ several amplification stages^13^,^14^,^15^,^16^ with intermediate pulse shaping^9^,^13^,^15^,^16^, whereas other schemes just rely on one amplification stage^17^,^18^,^19^. Regardless of the specific system design, the active fiber in the amplifier is the main temporal and spectral pulse shaping element owing to pump-dependent and spectrally varying gain distribution along the fiber, pulse interaction with co-propagating amplified spontaneous emission (ASE), dispersion, as well as non-resonant and resonant components of nonlinearities. Careful study of the fiber amplifier is mandatory for optimizing the overall system performance.

Since the equations governing the dynamics in pulsed FA are in general not possible to solve analytically, reliable simulations are required. FA are commonly modeled by using rate equations (RE) describing the amplified spontaneous emission (ASE) and gain along the fiber. For low intensity seed pulses and continuous wave (CW) operation most nonlinear effects can be neglected, making this approach sufficiently accurate^20^,^21^,^22^,^23^. However, when amplifying ultra-short pulses in FA, dispersive and nonlinear effects become more pronounced, making a pure RE approach incapable of modeling the complete dynamics. A common work-around to this problem is to assume a constant gain along the fiber when modeling the pulse evolution with the generalized nonlinear Schrödinger equation (GNLSE)^13^,^24^,^25^,^26^, analogous to the lumped-element approach used for modeling bulk solid state lasers and amplifiers. This approach is adequate for modeling steady state pulse regimes in mode-locked fiber lasers. However, the drawback is that factors that are generally important to consider in FA, such as spatially and wavelength dependent gain are neglected.

An early effort to include the effects described by the RE in the GNLSE model was based on using the susceptibility of a two-level system^27^. This approach lead to new terms in the GNLSE which described gain saturation and a finite gain bandwidth, but spatial gain variations were still not included. Sequentially solving transient RE and the GNLSE while updating the inversion level in between consecutive pulses has also been explored^20^. However, in order to satisfy the necessary boundary conditions, this method is only able to model cases when one pulse is propagating in the FA at a time, i.e. amplifiers operating at low repetition rates. Another approach, based on first solving the RE to predetermine gain parameters which are subsequently used in the GNLSE, has also been used to model FA^17^.

In contrast to any of the above mentioned approaches, the model presented here jointly solves the RE and the GNLSE and thus directly includes the interplay between dispersive, nonlinear and pump related effects. A similar approach was used by Chen *et al*.^28^. Both models assume a high repetition rate regime, which is where FA can be operated most efficiently as the ASE build up can be kept low, and can account for multiple pulses propagating in the fiber at the same time. However, their model was used to optimize chirped-pulse FA design only considering co-propagating configurations, whereas the model described here includes boundary conditions such that arbitrary pump configurations can be simulated. While this is already of great interest, as counter propagating FA are commonly used^14^,^15^,^16^,^18^,^26^, the model presented here also includes ASE, gain saturation and uses the inversion at each position to determine the gain for the GNLSE. Furthermore, the impact of the inversion level on the refractive index is also included.

This paper is structured as follows: first, the theoretical part introduces the necessary equations for the RE and the GNLSE. After that, a detailed account of the numerical approach illustrates how the two models were jointly solved in an iterative manner. Subsequently, the result part validates the model by comparing simulated and experimental data. In particular, the pump-power dependencies of pulse length, pulse spectra and output powers are compared for an in-house built FA as well as for a setup reported in the literature^14^. The comparisons are in good agreement, showing that the presented model is an accurate tool for designing high-repetition rate ultrashort FA systems. Finally, concluding remarks regarding the range of application of the described model as well as its limitations are given.

## Theory

The temporal dynamics of the energy level population in laser gain media can be modeled using RE^29^, which account for the absorption as well as stimulated and spontaneous emission of photons. By combining them with propagation equations for the photon flux at wavelengths of interest, the energy level populations can be determined throughout the laser gain media as well as the output spectrum and power.

Due to the simple two-multiplet energy level scheme in Yb-doped gain media it is convenient to solve RE for the combined population of the upper and lower laser multiplet. The relevant spectroscopic information is then contained in phenomenological (and measurable) absorption and emission cross sections, which are not dependent on the position in the fiber. In general, this approach is insufficient for other gain media, such as Er-doped fiber pumped at 980 nm where an additional equation for the population of the pump level is commonly required. However, this approach is sufficient for any rare-earth doped media pumped directly to the upper laser multiplet. With this simplification the spatially-dependent RE used in the combined model could be expressed as^20^


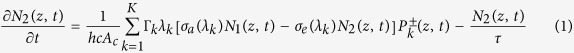



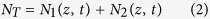



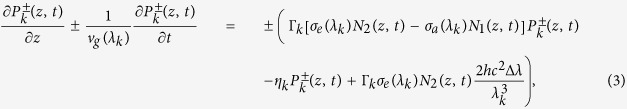


where *h* is Planck’s constant, *c* is the speed of light in vacuum, *z* is the position along the fiber, *t* is time, *v*_*g*_ is the group velocity, *N*_1_ and *N*_2_ are the effective lower and upper energy level populations. The wavelength-dependent absorption and emission cross-section for the transition are *σ*_*a*_ and *σ*_*e*_, respectively, and the upper level lifetime is *τ*. Δ*λ* is the wavelength resolution. The total doping concentration, *N*_*T*_, the core area, *A*_*c*_, as well as the background losses, *η*_*k*_, at wavelength *λ*_*k*_ were assumed to be constant throughout the fiber. The geometric overlap factors, Γ_*k*_, were updated along the fiber for the signal, as explained below, while it was kept constant for the pump. *P*_*k*_ denotes the power while the signs + and − correspond, respectively, to forward and backward propagating beams, therefore taking into account fiber-end reflections, bidirectional ASE and different pumping configurations. The sum appearing in equation (1) runs over all of the wavelengths in the considered spectral window.

Further simplification is possible by restricting the model to high-repetition rate FA, where it can be assumed that the population inversion, 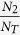, reaches steady state. Inserting equation (2) into equation (1) and solving for 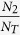 it is easy to see that at a given position, *z* along the fiber, the population inversion due to a CW monochromatic pump at the wavelength *λ*_*p*_ with a power *P*_*p*_(*z*) will grow as a saturating exponential function with a time constant of


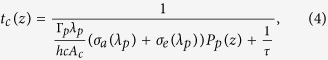


which is a measure of the position-dependent recovery time of *N*_2_. Obviously, the highest recovery rate, 1/*t*_*c*_, would be at the pumping-end of the fiber. For example, for a fiber with a core diameter of 20 μm and an inner cladding diameter of 400 μm pumped by 100 W at 976 nm this rate is approximately 20 kHz. For this estimation, data from Pask *et al*.^30^ was used for the upper level life time and the cross sections and Γ_*p*_ was approximated by the ratio of the core and cladding areas. If *t*_*c*_ is much greater than the time in between consecutive pulses, i.e. the repetition rate is much greater than 1/*t*_*c*_ at the pumping-end, the pump will not be able to recover *N*_2_ between the pulses. This implies that *N*_2_(*z*) will tend towards a steady state where the pump, the stimulated and the spontaneous emission balance each other. In this case, the time derivatives in equation (1) can be neglected. This was the approximation used in our model. Using this and solving equations (1 and 2) for *N*_2_ then gives


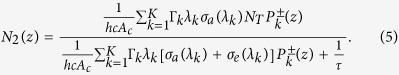


The time derivative in equation (3) is eliminated by casting the equation in the coordinate frame moving at the group velocity of the pulse. Moreover, the ASE in steady state will also be constant in time. Thus, under these conditions equation (3) reduces to


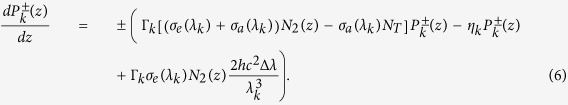


As the last term on the right hand side in equation (6) accounts for spontaneous emission, the position-dependent FA gain can be expressed as


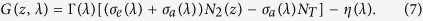


These equations are valid not only for high-repetition rate FA as defined above, but also in cases where the gain is saturated by ASE and when the amplified pulse intensity is substantially below the saturation intensity at low repetition rates. However, these regimes signify sub-optimum FA design and are therefore of little interest. For a low-repetition rate seed and efficient extraction of the energy from the population inversion the steady state condition cannot be assumed and, therefore, the full dynamics of the population inversion via equations (1 and 3) should be included in the model.

Propagation of the complex electric field envelope of the pulse, *A*, in fibers is well described by the GNLSE. When accounting for the effects of gain, dispersion, self-phase modulation (SPM), self-steepening and stimulated Raman scattering, it takes on the following form:


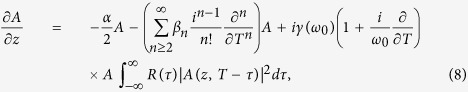


which is valid for spectral broadening of less than 20 THz^31^, where *ω*_0_ is the central angular frequency, *z* is the position along the fiber, *T* is the time in the frame moving at the pulse’s group velocity, *α* is the absorption (gain) coefficient, *β*_*n*_ are the Taylor expansion coefficients for the mode-propagation constant 
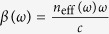
, where n_eff_ (*ω*) is the effective refractive index at the angular frequency *ω*. The nonlinear response function, *R*(*τ*), is given by


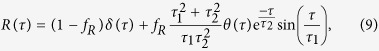


where *f*_*R*_ is the fractional contribution of the Raman response, *θ*(*T*) is the Heaviside step function, *τ*_1_ and *τ*_2_ are parameters to adjust the function to the Raman gain spectrum. The first term is the instantaneous Kerr response and the second term is the delayed response of a damped Raman oscillator. This model of the Raman response has been used to study pulses as short as 8 fs^32^. In our simulations, the Raman process parameters were: *f*_*R*_ = 0.18, *τ*_1_ = 12.2 fs and *τ*_2_ = 32 fs^31^. The nonlinear parameter is defined in the usual way, *γ*(*ω*_0_) = 3*ω*_0_ Re 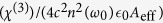, where *A*_eff_ is the effective mode area, 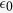 is the dielectric permittivity of free space and *χ*^(3)^ is the third order electronic susceptibility. Therefore, *γ*(*ω*_0_) has units of W^−1^m^−1^ and the field envelope amplitude in equation (8) is defined as 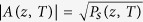, where *P*_*S*_(*z*, *T*) is the instantaneous power of the pulse.

When considering ultrashort pulses in broadband gain media, such as Yb-doped fibers, the wavelength dependence of the cross-sections in equation (7) has to be taken into account. Therefore the net absorption will be wavelength dependent, which has to be reflected in the GNLSE. This is accounted for by introducing an additional sum in equation (8) containing the Taylor expansion coefficients of the angular frequency dependent net absorption *α*(*z*, *ω*), in the same manner as the sum containing the *β*_*n*_-terms is introduced^31^. This gives the following equation:


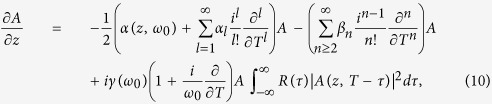


where *α*(*z*, *ω*_0_) is the absorption at the central angular frequency and *α*_*l*_ are the Taylor expansion terms of the angular frequency dependence of *α*(*z*, *ω*).

In addition, gain saturation can also lead to pulse distortions. This effect was included in the GNLSE by multiplying the term *α*(*z*, *ω*_0_) with an exponential describing the accumulated energy of the pulse, 
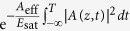
, where *E*_sat_ = *A*_eff_*hc*/[*λ*_0_Γ(*σ*_*e*_(*λ*_0_) + *σ*_*a*_(*λ*_0_))] is the saturation energy and *λ*_0_ is the central wavelength of the pulse^20^.

It is well-known that a Lorentzian absorption line has an associated impact on the refractive index. Considering an electronic transition from level i to j in Yb, the contribution to the refractive index can be described by^33^


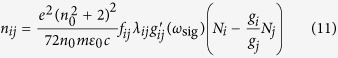


where *e* is the electron charge, *n*_0_ the refractive index of the host, *m* is the electron mass, 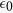 is the free space permittivity, *f*_*ij*_ is the oscillator strength, *λ*_*ij*_ is the transition line center, 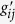 is the lineshape, *ω*_sig_ is the angular frequency of the incident light, *N*_*i,j*_ are the population densities and *g*_*i,j*_ are the degeneracy factors. Using the relation between oscillator strength, absorption cross-sections and the Einstein coefficients^34^, equation (11) can be re-expressed as


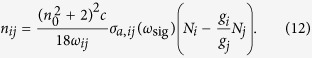


This equation is to be summed over all transitions in order to get the net contribution. However, given the limited amount of extractable information in cross-section data, we instead used the available cross-sections in conjunction with the mean transition frequency (replacing *ω*_*ij*_) to model the impact of transitions from *N*_1_ to *N*_2_. *g*_1_, *g*_2_ and the UV-level parameters were set to the values given in the paper by Arkwright *et al*.^33^. The UV-level contributions were evaluated by their asymptotic values^35^ as the wavelengths considered in this work are far from their resonances. *n*_0_ is set by using the unpumped version of equation (12), the Sellmeier equation for fused silica^36^ and the fiber NA. Once obtained, *n*_0_ is used to evaluate equation (12) which is then added to the passive core index. The resulting refractive index and the one for fused silica is then used to solve the standard eigenvalue equation for a step-index fiber^31^ to determine the propagation constant of the fundamental mode, for all the wavelengths in the considered spectral window at different levels of inversion. The dispersion is determined by subtracting the value of the propagation constants and their first order derivatives at the seed’s central wavelength, in order to conform with the notation used in equation (10). The propagation constants are also used to determine the effective mode area and hence the nonlinear parameter, *γ*, as well as the signal overlap factor, calculated as 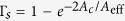, at different levels of inversion and for different wavelengths.

## Joint solution of the RE and the GNLSE

The RE, equations (5 and 6), and the GNLSE, equation (10), are coupled through the absorption *α*(*z*, *ω*), which is simply the negative of the gain, given in equation (7). Thus, at each position in the fiber, the value of *N*_2_ is first determined, using equation (5), and is thereafter used to determine *α*(*z*, *ω*) as well as the dispersion, the nonlinear parameter and the geometric overlap factor corresponding to the current level of inversion. These parameters are then used in the GNLSE, to propagate the pulse, and in the propagation equations (6), to propagate the pump and the ASE powers in both directions. The propagation equations are evaluated by applying the standard fourth-order Runge-Kutta (RK4) scheme for first-order ordinary differential equations (ODE).

The GNLSE is solved using a scheme called the fourth-order Runge-Kutta in the interaction picture method (RK4IP)^37^, which alternates between the Fourier domain, to evaluate dispersive effects, and the time domain, to evaluate nonlinear effects. Using this method, the GNLSE is expressed by using the following dispersive and nonlinear operators, respectively,


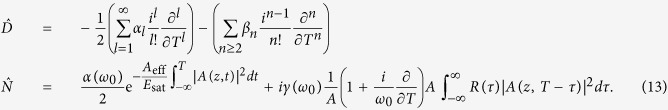


Thus, the GNLSE takes on the following form


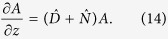


By transforming this equation into an interaction picture it can be re-expressed as a first-order ODE, which can be solved with arbitrary accuracy by applying methods of perturbation theory, as used in quantum mechanics. However, for computational efficiency the RK4 scheme was used instead. This method then gives an accuracy proportional to the fourth order of the used step-size, as opposed to commonly used split-step Fourier methods which are accurate to the second and third order^37^. A summary of this procedure is detailed in the supplementary information part of this paper.

Now, Equation (6) only considers average powers, i.e. it does not contain temporal information in the coordinate frame co-moving with the pulse. It is therefore necessary to appropriately relate the average pulse power used in the RE to the electric field amplitude *A*(*z*, *T*) used in the GNLSE. Thus, when evaluating Equation (5) at each step along the fiber, the amplitude of the pulse’s electric field envelope is renormalized using


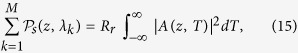


where 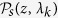 are the Fourier components of the pulse’s average power spectrum (converted to wavelength constituents), *M* is the total number of constituents in the power spectrum within the considered spectral window and *R*_*r*_ is the pulse repetition rate. Using such normalization allows, if necessary, to use different discretization scales in the numerical solutions of the RE and the GNLSE. In this work, an identical discretization in the wavelength (frequency) domain for the RE and the GNSLE was chosen for convenience. The discretization is in principle determined by the requirement of well-resolved pulse amplitude and phase variations as well as having sufficient margin to avoid Fourier window spillover, which would give numerical artifacts. The latter can also be mitigated by absorbing boundaries of the spectral window, provided that power calibration is performed at each step along the fiber.

### Propagation schemes

Longitudinal evolution profiles for pump, ASE and seed pulse powers can then in principle be determined by iteratively solving the equations with the previously described joint approach. However, a direct solution would require prior knowledge of the initial values for all these signals. In general, this is not available, especially if pump and seed signals are launched at different fiber ends or if feedback from the fiber end facets are to be considered. Such boundary value problems can be reduced to initial value problems by applying a shooting-method, as e.g. outlined by Zeil and Laurell^21^. This method solves the equations iteratively going back and forth between the fiber ends and imposing boundary conditions upon reaching the fiber ends. In this way, the boundary values are applied as initial values for each propagation.

**Figure 1:**
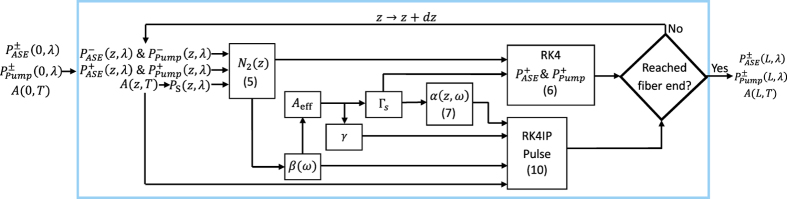
Illustration of the forward propagation, from *z* = 0 to *z* = *L*, scheme used in each iteration. The numbers in parentheses denote the equations being used.

The full algorithm is based on two propagation schemes, one from the end where the seed is injected, *z* = 0, to the end where the amplified pulse is exiting, *z* = *L*, and one in the opposite direction. The propagation scheme from *z* = 0 to *z* = *L* is illustrated in Fig. 1. At each position of the fiber the pulse, the ASE and the pump are used in equation (5) to determine *N*_2_. This value is used in the propagation equations, after using it to determine Γ_*s*_, for the forward propagating (+signs in equation (6)) pump and ASE to propagate them to the next position in the fiber. The backward propagating pump and ASE (−signs in equation (6)) values are not updated in this direction, due to numerical stability, instead their values from the previous *z* = *L* to *z* = 0 propagation are used. The *N*_2_-value is also used to determine *α*(*z*, *ω*), *β*(*ω*) and *γ* which are then used in the GNLSE to propagate the pulse to the next position in the fiber. This is repeated until reaching *z* = *L*.

**Figure 2:**
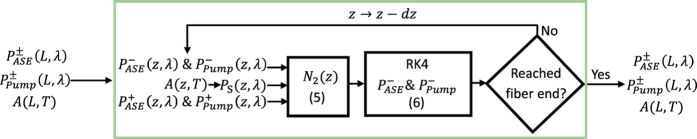
Illustration of the backward propagation, from *z* = *L* to *z* = 0, scheme used in each iteration. The numbers in parentheses denote the equations being used.

In the propagation from *z* = *L* to *z* = 0, shown in Fig. 2, the forward propagating signals are not updated. Instead, the values of the forward propagating signals from the previous propagation from *z* = 0 to *z* = *L* are used when determining the value of *N*_2_ at a specific position. This value is then used to propagate the backward pump and ASE to the next position. This is repeated until reaching *z* = 0.

Upon reaching the fiber ends, the following boundary conditions are applied to the ASE and pump


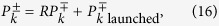


where *R* is the reflection at the fiber end and 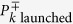 are launched powers. The power of the seed pulse is reset to its launched value at *z* = 0, i.e. the reflected signal is neglected. This is justified in single pass FA as the reflection at the exiting fiber end is several orders of magnitude smaller than the launched one, usually due to angle cleaving. Nonetheless, the reflected pulse can easily be included by propagating the reflected part of *A*(*L*, *T*) with the GNLSE in the *z* = *L* to *z* = 0 propagation scheme. The reflected pulse must then also be included in the propagation from *z* = 0 to *z* = *L* and be related to its wavelength constituents to be included when calculating *N*_2_.

The propagation schemes and boundary conditions are run between the fiber ends until the output power between the last two iterations has not changed more than some predetermined value. The convergence process is characterized by a gradually decreasing alternation of over- and underestimation of the output power between consecutive iterations.

**Figure 3:**
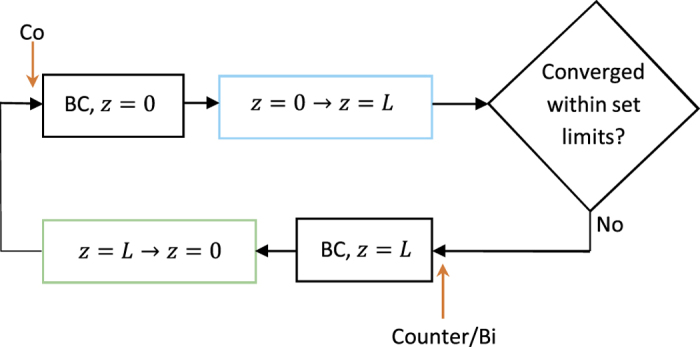
Schematic illustration of the overall scheme of the numerical model. The red arrows indicate where the algorithm is initiated for co-, counter and bi-directionally pumped configurations. BC denotes boundary conditions.

Depending on the pump configuration, the algorithm is initiated differently to speed up the convergence. For counter- and bi-directionally pumped setups, the boundary conditions at *z* = *L* and the propagation scheme from *z* = *L* to *z* = 0 are run first to set up an inversion level for the pulse. While for co-propagating setups, the boundary conditions at *z* = 0 and the propagation scheme from *z* = 0 to *z* = *L* are run first. This is illustrated in Fig. 3, where the red arrows show where the algorithm is initiated depending on the pumping technique.

## Results

Carrying out accurate simulations requires reliable gain fiber-specific parameters, primarily cross-section data. We therefore used absorption and emission cross-sections based on reported experimental data. Furthermore, the obtained frequency and inversion dependencies of *α* and *β* were directly applied in the Fourier domain when using the RK4IP, as this proved to give more accurate results than using the Taylor expansion coefficients, i.e. *α*_*l*_ and *β*_*n*_, in equation (10). Also, the above mentioned spectral windows spanned from 1000–1100 nm as this proved broad enough to accommodate pulse and ASE spectra, while avoiding numerical spillover effects.

The remainder of this section discusses comparison of simulations using our model and experimental results. Two different FA setups were used: one that was built in-house and one reported in the literature which provided sufficient data for a relevant comparison^14^. The upper level lifetime, *τ*, was set to 840 μs^30^ in both simulations. A summary of the fiber specific parameters used in the simulations can be found in Supplementary Table S1.

**Figure 4:**
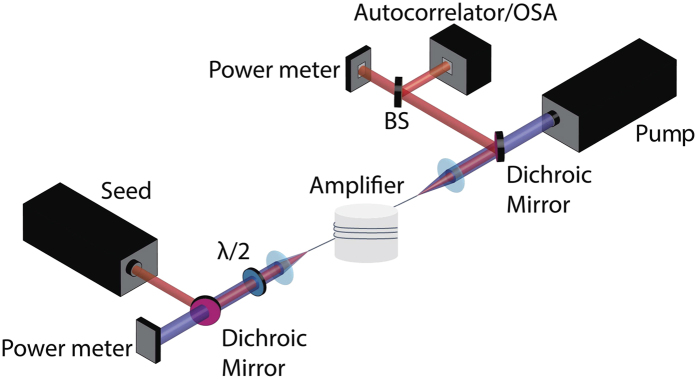
Schematic representation of the MOPA setup used to validate the model. BS, *λ*/2 and OSA denote beam splitter, half-wave plate and optical spectrum analyzer respectively.

As a first test, a counter propagating MOPA setup was built for validation. The setup is illustrated in Fig. 4 and consisted of a polarization maintaining double clad 5.7 m long Yb-doped fiber from NUFERN Company with a small signal absorption of 0.5 dB/m at 915 nm and core/inner cladding diameter of 20/400 *μ*m. The amplifier was pumped by a volume Bragg grating stabilized laser diode at 976 nm. The seed was a Yb:KYW oscillator mode-locked using a quantum well saturable absorber generating transform-limited 173 fs pulses at a repetition rate of 212 MHz centered around 1041 nm. Both fiber ends were angle-cleaved to prevent back reflections. A dichroic mirror (DM) was used to separate the amplified pulses from the incident pump beam on one end and a second DM was used to separate the unabsorbed pump from the seed on the other end. The average power of the seed was 375 mW and the launched pump power was varied from 3 to 38 W, the coupling efficiencies were 80% and 90% for the seed and the pump, respectively. The orientation of the seed’s polarization was controlled with a half-wave plate. The pump overlap factor was approximated by Γ_*p*_ = *A*_*c*_/*A*, where *A* is the cladding area. Γ_*s*_ was determined from the calculated mode-field areas which were scaled to match the measured modefield area of 410 μm^2^ in the unpumped fiber. The nonlinear parameter, *γ*, was also determined by using the scaled mode areas and a nonlinear refractive index of *n*_2_ = 3.07 · 10^−20^ m^2^W^−1^, related to the third order electronic susceptibility by 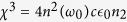
38. The value of *n*_2_ as well as the cross-section data for the fiber were supplied by NUFERN Company.

**Figure 5:**
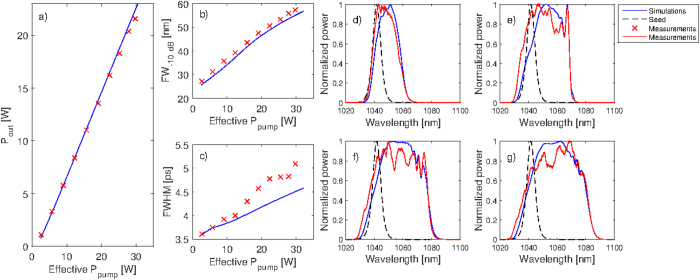
Simulated and measured (**a**) output power, (**b**) spectral widths at −10 dB and (**c**) full width at half maximum (FWHM) of the autocorrelation traces. The four right-most plots show spectral comparisons at output powers of (**d**) 1.1 W, (**e**) 8.4 W, (**f**) 16.2 W and (**g**) 20.3 W. The dashed curves show the measured seed spectrum.

Studying the numerical and experimental results shown in Fig. 5, it is clear that the output power characteristic, plotted versus effective pump (launched minus unabsorbed), is well predicted. It is also evident that the simulated spectral widths follow an essentially identical trend as the measured ones with a constant offset of just 2 nm. The measured and simulated pulse durations seem to follow a similar trend but with a constant offset of about 0.25 ps. These offsets are mainly attributed to the lack of knowledge of the exact dispersion, since the one used for our simulations was based on the refractive index for pure silica modified by the cross-section data. In reality, the dispersion is also affected by dopants used to increase/decrease the core/cladding refractive indexes. Therefore, a slight offset is to be expected. Comparisons of the simulated and measured autocorrelation traces are given in Supplementary Fig. S2.

Studying the spectra, shown in Fig. 5, shows quite good correspondence between the experiments and the simulations although there are some discrepancies in the positions and magnitude of some of the spectral features. These discrepancies, which are related to the differences in the pulse widths, are attributed to deviations between the actual and the literature values that were used for some of the parameters in the simulations, mainly the emission and absorption cross-section spectra. The model correctly predicts the shift of the spectra from 1041 nm to around 1060 nm, due to the gain asymmetry, something that cannot be predicted without the wavelength dependent gain.

As a second evaluation of the model we used data from Zhao *et al*.^14^, who demonstrated generation of 150 W of average power from a 2.5 ps oscillator with a repetition rate of 50 MHz after passage through three amplification stages. The first preamplifier was a co-propagating highly Yb-doped single-clad fiber, while the second preamplifier and the main amplifier were counter propagating Yb-doped rod-type fibers. As the only indication of the seed pulses’ chirp was a time-bandwidth product of 0.37, slightly exceeding the transform-limit. Using our model and comparing the obtained spectra with the experimentally reported ones, we could actually determine the sign of the input pulses’ chirp.

Hyperbolic secant pulses with negative and positive chirps corresponding to the measured time-bandwidth product were set up and propagated through the amplifiers. The pump/signal mode-field overlap were adjusted to match the output versus pump power in the amplifiers. Similarly, the values of *n*_2_ were adjusted to broaden the pulse spectra in the amplifiers to acquire the presented widths. The final values used in the simulations were *n*_2 preamplifier 1_ = 2.6 · 10^−20^ m^2^W^−1^, *n*_2 preamplifier 2_ = *n*_2 preamplifier_ = 1.2 · 10^−20^  m^2^W^−1^. The cross-sections were taken from Pask *et al*.^30^.

**Figure 6:**
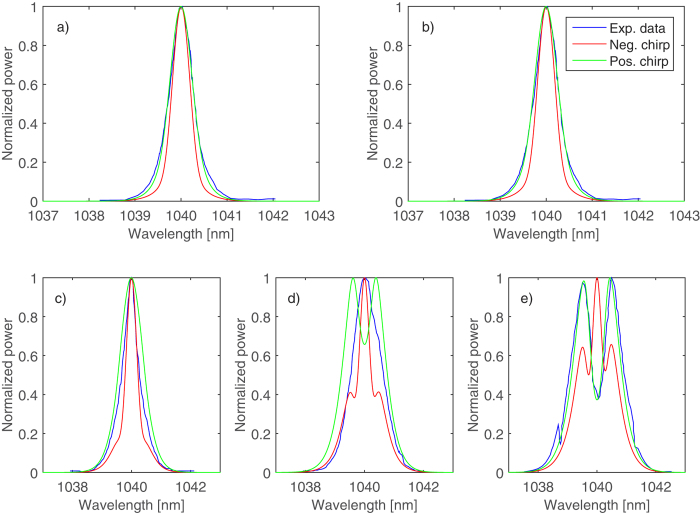
Comparison of experimental and simulated spectra in the (**a**) first and (**b**) second preamplifier and in the main amplifier at output powers of (**c**) 25 W, (**d**) 75 W and (**e**) 100 W. The blue curves denote the experimental data^14^ while the red and the green curves denote the simulated spectra using negatively and positively chirped seed pulses, respectively.

Figure 6 shows the generated spectra of linearly chirped pulses compared to the experimentally measured spectra at output powers where no transverse modal instabilities were present, as the GNLSE used in this work assumes single-mode operation. It is readily seen that the spectral evolution of the positively chirped pulses is in better agreement with the experimental data than the negatively chirped pulses’ evolution. Clearly the seed pulses with negative chirp would experience initial spectral compression due to the action of SPM before the spectral broadening takes place at higher peak powers. For positively chirped pulses, the spectral compression does not happen and the broadening produces a well-known dip in the center of the pulse spectrum. The model predicts an earlier appearance of such a dip as compared to the experimental data (Fig. 6d) which we tentatively attribute to the presence of the second order negative chirp in the experimental seed pulses, whereas we only considered a linear chirp.

## Conclusion

A conceptually simple method was developed for solving the RE and the GNLSE together in a FA in the case of a high repetition rate source and a CW pump. Based on this method, two propagation schemes were set up that could be combined to simulate arbitrarily pumped fiber based MOPA setups. As the model is based on RK4 schemes when solving both the GNLSE and the RE, it has a global accuracy proportional to the fourth order of the step-size.

In order to validate the model, a counter propagating fiber based MOPA was set up whose characteristics were in good agreement with the simulations. Additionally the model was employed to investigate another experimentally realized fiber based MOPA from the literature^14^ and provided results that were in good correlation. Thus, we believe that this model is well suited for studying and understanding the pulse evolution in fiber based MOPA that are operated at high repetition rates and in single transverse mode. The model could therefore serve as a valuable tool when optimizing the design of these types of systems, for instance in terms of pulse compressibility and maximizing output powers by appropriate choice of pump configuration, wavelength regimes, fiber lengths etc.

Using a transversely resolved model could improve the results and it would also allow the description of multi-mode amplification. However, this would be at the expense of a substantial increase in computation time.

Although the accuracy of the model was demonstrated in the context of Yb-doped FA it can also simulate other types of rare-earth doped ultrafast FA, given reliable spectroscopic data for the absorption and emission cross-sections and adjusting the RE correctly. The same scheme is also readily adaptable for simulation of mode-locked fiber lasers by inclusion of appropriate mechanisms for saturable loss.

## Additional Information

**How to cite this article**: Lindberg, R. *et al*. Accurate modeling of high-repetition rate ultrashort pulse amplification in optical fibers. *Sci. Rep.* **6**, 34742; doi: 10.1038/srep34742 (2016).

## Supplementary Material

Supplementary Information
