# Supplementary material for: Accurate modeling of high-repetition rate ultrashort pulse amplification in optical fibers
Source: Sci Rep. 2016 Oct 7;6:34742. doi: 10.1038/srep34742 (PMC5054424; doi:10.1038/srep34742)
Supplement: Supplementary Information [file srep34742-s1.pdf]

Accurate modeling of high-repetition rate ultrashort pulse amplification in optical fibers

Robert Lindberg[1,*], Peter Zeil[1], Mikael Malmström[1], Fredrik Laurell[1], and Valdas Pasiskevicius[1]

[1]Royal Institute of Technology, Applied Physics, Stockholm, 106 91, Sweden
[*]rl@laserphysics.kth.se

Supplementary information

Fourth order Runge-Kutta in the interaction picture algorithm

Expressing the GNLSE like:

$$\frac{\partial A}{\partial z} = \left(\hat{D} + \hat{N}\right) A. \tag{S1}$$

allows for the transformation to an interaction picture by defining the interaction amplitude as $A^I = \mathrm{e}^{-(z-z')\hat{D}} A$, where z' is the separation distance between the normal and the interaction picture, and the interaction nonlinear operator as $N^I = \mathrm{e}^{-(z-z')\hat{D}} \hat{N} \mathrm{e}^{(z-z')\hat{D}}$. This yields the following ODE:

$$\frac{\partial A^I}{\partial z} = N^I A^I \tag{S2}$$

Applying the standard RK4 scheme to this equation and setting $z' = \Delta z/2 + z$ gives:[1]

$$\begin{aligned}
A^I(z_i,T) &= \mathrm{e}^{\frac{\Delta z}{2}\hat{D}} A(z_i,T) \\
k_1 &= \Delta z \mathrm{e}^{\frac{\Delta z}{2}\hat{D}} \hat{N}\left(A(z_i,T)\right) A(z_i,T) \\
k_2 &= \Delta z \hat{N}\left(A^I(z_i,T) + k_1/2\right)\left[A^I(z_i,T) + k_1/2\right] \\
k_3 &= \Delta z \hat{N}\left(A^I(z_i,T) + k_2/2\right)\left[A^I(z_i,T) + k_2/2\right] \\
k_4 &= \Delta z \hat{N}\left(\mathrm{e}^{\frac{\Delta z}{2}\hat{D}}[A^I(z_i,T) + k_3]\right)\mathrm{e}^{\frac{\Delta z}{2}\hat{D}}\left[A^I(z_i,T) + k_3\right] \\
A(z_i + \Delta z, T) &= \mathrm{e}^{\frac{\Delta z}{2}\hat{D}}\left[A^I(z_i,T) + k_1/6 + (k_2 + k_3)/3\right] + k_4/6.
\end{aligned} \tag{S3-S8}$$

The derivatives in the dispersion exponential appearing in the RK4IP are bypassed by using Fourier analysis, i.e. the functions that the dispersion exponential are to operate on are Fourier transformed and multiplied by the frequency dispersion exponential, the products are then inversely transformed.

Discretization of the fiber

Given well enough resolved time and spectral profiles of the pulse, the discretization of the length of the fiber is the main limiting factor for accurately solving the GNLSE when using the RK4IP method. An adaptive step size conserving the classical photon number of the pulse between consecutive steps along the fiber[2] was implemented to address this. However, a two dimensional interpolation, over position and wavelength, for the quantities being updated in the backward propagation, i.e. from $z = L$ to $z = 0$, had to be implemented in the forward propagation, as the number of steps along the fiber changes in this direction.

Simulation parameters

The model relies on several fiber parameters as explained in the main text, the values that were used in the presented results are summarized in the table below.

	In-house FA		Zhao et al.	
Parameter	Value	Source	Value	Source
α_{ss}	-0.5_{915} dBm^{-1}	NUFERN	-700^{a}_{976} / $-30^{b,c}_{976}$ dBm^{-1}	CorActive[a] / NKT Photonics[b,c]
τ	840 μs	Pask et al.[3]	840 μs	Paske et al.[3]
L	5.7 m	Measured	0.1^{a} / 0.36^{b} / 0.55^{c} m	Zhao et al.[4]
A_c	363 μm^2	NUFERN	52.8^{a} / $785^{b,c}$ μm^2	CorActive[a] / NKT Photonics[b,c]
A	0.128 mm^2	NUFERN	0.0123^{a} / $0.0638^{b,c}$ mm^2	CorActive[a] / NKT Photonics[b,c]
A_{eff}	410 μm^2	Measured	19.6^{a} / $4500^{b,c}$ μm^2	CorActive[a] / NKT Photonics[b,c]
η_{signal}	1 dBkm^{-1}	Senior[5]	1 dBkm^{-1}	Senior[5]
η_{pump}	1.5 dBkm^{-1}	Senior[5]	1.5 dBkm^{-1}	Senior[5]
n_2	$3.07 \cdot 10^{-20}$ m^2W^{-1}	NUFERN	$2.6 \cdot 10^{-20\,a}$ / $1.2 \cdot 10^{-20\,b,c}$ m^2W^{-1}	Fitted
f_R	0.18	Agrawal[6]	0.18	Agrawal[6]
τ_1	12.2 fs	Agrawal[6]	12.2 fs	Agrawal[6]
τ_2	32 fs	Agrawal[6]	32 fs	Agrawal[6]
R_0	$8 \cdot 10^{-4}$	Calculated	$8 \cdot 10^{-4}$	Fitted
R_L	$8 \cdot 10^{-4}$	Calculated	$8 \cdot 10^{-4}$	Fitted

Supplementary Table S1. Summary of the simulation parameters. The subscripts on the small signal absorption values, α_{ss}, denote the wavelengths at which they are valid. The superscripts a, b and c correspond respectively to the values used for preamplifier 1, preamplifier 2 and the main amplifier, if they differed. NUFERN, CorActive and NKT Photonics listed under sources refer to data available from the vendors.

The small signal absorptions listed in the table were used to estimate the doping concentration, N_T. This can be done by solving equation (3) in the main text for the pump and assuming that all ions are in the ground-state, i.e. $N_2 = 0$ and $N_1 = N_T$. Doing so gives

$$N_T = -\frac{\ln\left(10^{\frac{\alpha_{ss}}{10}}\right)}{\Gamma_p \sigma_{\text{abs}}(\lambda_{\text{pump}})}, \tag{S9}$$

where λ_{pump} is the pump wavelength.

Autocorrelation traces

The autocorrelation traces were obtained using Autocorrelator Model 409 from Spectra Physics connected to an oscilloscope. The comparison between the simulated and measured autocorrelation traces are shown in the figure below.

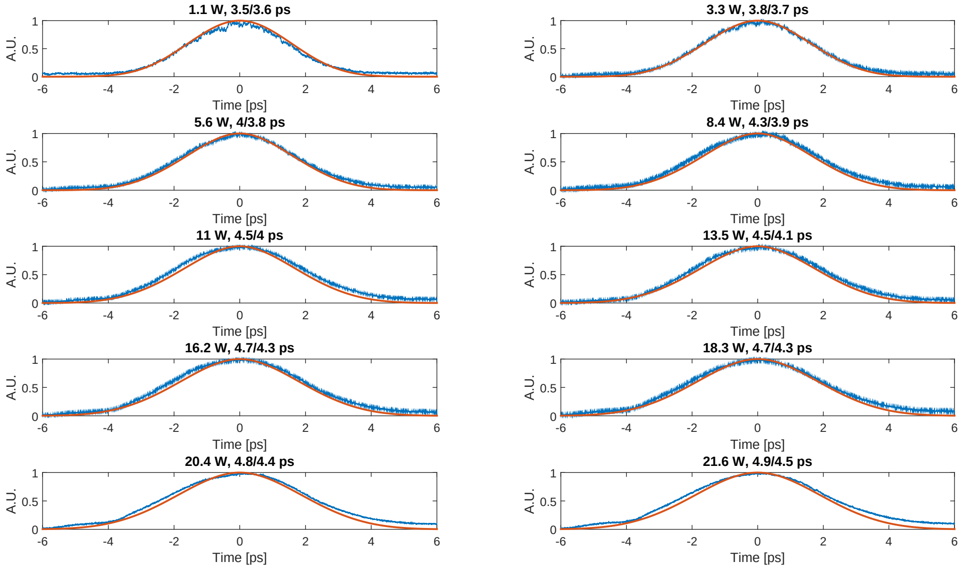

Supplementary Figure S1. Comparison between the measured, blue, and simulated, red, autocorrelation traces. The numbers in the title above each graph represent "output power, measured/simulated FWHM".

References

1. Hult, J. A fourth-order Runge - Kutta in the interaction picture method for simulating supercontinuum generation in optical fibers. *J. Lightw. Technol.* **25**, 3770–3775 (2007).

2. Heidt, A. M. Efficient adaptive step size method for the simulation of supercontinuum generation in optical fibers. *J. Lightw. Technol.* **27**, 3984–3991 (2009).

3. Pask, H. *et al.* Ytterbium-doped silica fiber lasers: versatile sources for the 1-1.2 μm region. *IEEE J. Sel. Topics Quantum Electron.* **1**, B63–B92 (1995).

4. Zhao, Z., Dunham, B. M. & Wise, F. W. Generation of 150 W average and 1 MW peak power picosecond pulses from a rod-type fiber maser oscillator power amplifier. *J. Opt. Soc. Am. B* **31**, 33–36 (2014).

5. Senior, J. M. *Optical Fiber Communications Principles and Practice*, chap. 3.3.1, 91 (Pearson Education, 2009).

6. Agrawal, G. P. *Nonlinear Fiber Optics 4th edn*, chap. 2 & 11, 29-40 & 424-425 (Academic Press, 2007).
